# Lack of ABCG2 Leads to Biventricular Dysfunction and Remodeling in Response to Hypoxia

**DOI:** 10.3389/fphys.2017.00098

**Published:** 2017-02-21

**Authors:** Bence M. Nagy, Chandran Nagaraj, Bakytbek Egemnazarov, Grazyna Kwapiszewska, Rudolf E. Stauber, Alexander Avian, Horst Olschewski, Andrea Olschewski

**Affiliations:** ^1^Division of Pulmonology, Department of Internal Medicine, Medical University of GrazGraz, Austria; ^2^Ludwig Boltzmann Institute for Lung Vascular ResearchGraz, Austria; ^3^Institute of Physiology, Medical University of GrazGraz, Austria; ^4^Division of Gastroenterology and Hepatology, Department of Internal Medicine, Medical University of GrazGraz, Austria; ^5^Institute for Medical Informatics, Statistics and Documentation, Medical University of GrazGraz, Austria

**Keywords:** ABC transporters, right ventricular function, hypoxia, diastolic dysfunction, ventricular fibrosis, pulmonary hypertension

## Abstract

**Aims:** The ATP-binding cassette (ABC)G2 transporter protects the heart from pressure overload-induced ventricular dysfunction but also protects cancer cells from chemotherapeutic agents. It is upregulated in the myocardium of heart failure patients and clears hypoxia-induced intracellular metabolites. This study employs ABCG2 knockout (KO) mice to elucidate the relevance of ABCG2 for cardiac and pulmonary vascular structure and function in chronic hypoxia, and uses human primary cardiac fibroblasts to investigate the potential role of ABCG2 in cardiac fibrosis.

**Methods and results:** ABCG2 KO and control mice (*n* = 10) were subjected to 4 weeks normoxia or hypoxia. This allowed for investigation of the interaction between genotype and hypoxia (GxH). In hypoxia, KO mice showed pronounced right (RV) and left (LV) ventricular diastolic dysfunction. Compared to normoxia, end-diastolic pressure (EDP) was increased in control vs. KO mice by +1.1 ± 0.3 mmHg vs. +4.8 ± 0.3 mmHg, *p* for GxH < 0.001 (RV) and +3.9 ± 0.5 mmHg vs. +11.5 ± 1.6 mmHg, p for GxH = 0.110 (LV). The same applied for myocardial fibrosis with +0.3 ± 0.1% vs. 1.3 ± 0.2%, p for GxH = 0.036 (RV) and +0.06 ± 0.03% vs. +0.36 ± 0.08%, *p* for GxH = 0.002 (LV), whereas systolic function and capillary density was unaffected. ABCG2 deficiency did not influence hypoxia-induced pulmonary hypertension or vascular remodeling. In line with these observations, human cardiac fibroblasts showed increased collagen production upon ABCG2 silencing in hypoxia (p for GxH = 0.04).

**Conclusion:** Here we provide evidence for the first time that ABCG2 membrane transporter can play a crucial role in ventricular dysfunction and fibrosis in hypoxia-induced pulmonary hypertension.

## Introduction

Right ventricular (RV) failure is the main cause of premature death of patients with pulmonary hypertension (PH) (Bogaard et al., 2009; Paulin et al., 2015). Despite a growing body of evidence on molecular mechanisms of RV failure, there are abundant unknown pathways and factors needed to be identified. RV dysfunction is commonly seen as a consequence of chronic RV pressure overload and a developing imbalance of RV oxygen demand and supply with neurohormonal activation and abnormal energy metabolism (Sano et al., 2007; Bogaard et al., 2009; Tuder et al., 2012; Voelkel et al., 2012; Vonk-Noordegraaf and Westerhof, 2013; Paulin et al., 2015). But there are also structural changes with myocardial fibrosis as the most striking feature that is strongly associated with RV dysfunction and prognosis.

ABC transporters are active transmembrane proteins capable to move a wide variety of substrates across extra- and intracellular membranes thereby modulating cell metabolism and cellular toxicity (Dean et al., 2001). Recent study shows, that knock out or inhibition of ABCC4 transporter (also known as MRP4) prevents and reverses hypoxia-induced pulmonary hypertension in mice (Hara et al., 2011). Similarly to ABCC4, another ABC transporter family member, the ABCG2 is transcriptionally regulated by hypoxia-inducible transcription factor (HIF) and exports endogenous toxic metabolites that are induced by chronic hypoxia, thereby preventing their intracellular retention (Jonker et al., 2002; Krishnamurthy et al., 2004; Zhou et al., 2005). Loss of ABCG2 results in an accumulation of porphyrins, and haeme degradation products and portends poor survival under hypoxia (Krishnamurthy et al., 2004). The expression of ABCG2 transporter was predominantly localized to the capillary endothelial cells, and cardiomyocytes in the heart (Solbach et al., 2008; Higashikuni et al., 2010). In heart failure, the left ventricular expression of the ABCG2 transporter is strongly elevated and correlates with atrial natriuretic peptide (ANP) (Solbach et al., 2008). In addition, ABCG2 protects against pressure overload-induced cardiac hypertrophy and left heart failure (Higashikuni et al., 2012) and is cardioprotective after myocardial infarction (Higashikuni et al., 2010). These findings suggest that ABCG2 is an important protective factor for the myocardium, particularly under conditions of chronic myocardial hypoxia like ischemic heart disease or lung disease with hypoxemia.

In the clinical setting, ABCG2 is also known as Breast Cancer Resistance Protein (BCRP), as one of the major factors causing drug resistance in cancer cells (Szakacs et al., 2006; Wu et al., 2008). ABCG2 transports many different substrates, providing resistance against multiple chemotherapeutic agents. Therefore, ABCG2 has been considered as an important target in cancer treatment to improve sensitivity for chemotherapy (Breedveld et al., 2006; Hardwick et al., 2007) and ABCG2 inhibitors have been tested in clinical studies(Kruijtzer et al., 2002; Kuppens et al., 2007; Molina et al., 2008). Such new drug developments necessitate a thorough investigation of the role of the molecular target not only under physiological but also under stress conditions which are commonly present in cancer patients with their main comorbidities.

In this study, we explored the role of ABCG2 in hypoxia-induced remodeling of the right ventricle. We provide evidence that under chronic hypoxia, loss of ABCG2 leads to biventricular fibrosis with diastolic dysfunction, without affecting RV afterload, capillary density or hypoxia-induced pulmonary vascular remodeling.

## Methods

### Animals

Male ABCG2 global knockout (KO) mice (FVB/N) were purchased from Taconic Europe (Laven, Denmark). We applied the chronic hypoxic mouse model, a routinely used method in various research fields (Gamboa and Andrade, 2010; Crnkovic et al., 2016), which allows the investigation of the effects of hypoxia. The experimental condition of 10% oxygen corresponds to a height of about 5,000 m. For the hypoxia-induced pulmonary hypertension group 10–12 week old mice (10 wild type (WT) and 11 KO per group) were placed in normobaric 10% hypoxic chambers [fraction of inspired oxygen (FiO_2_) of 0.10] for 4 weeks. Control mice were kept in normobaric normoxia (FiO_2_ of 0.21). Oxygen concentration was monitored and maintained continuously using the OxyCycler system (BioSpherix, Lacona, NY, USA). ABCG2 KO mice and WT animals were kept under conventional conditions on chow-fed diet. Chambers were opened twice per week for feeding and cleaning. All experiments were approved by the Federal Ministry of Science and Research (Austria), according to national regulations (Austrian Ministry of Education, Science and Culture, BMWF-66.010/0062-II/3b/2012).

### Haemodynamic measurements

Heart catheterization was performed under constant inhalation of 2% isoflurane-oxygen narcosis, using the closed chest technique through a small incision on the submandibular area, as described previously (Crnkovic et al., 2011). The right ventricle (RV) was catheterized via the right jugular vein and pressures were monitored continuously in order to determine the position of the catheter. After data collection from the right heart, a catheter was inserted into the right carotid artery where blood pressure was measured, and then advanced into the left ventricle where pressures were also recorded. Left ventricular pressure measurements—demanding an additional vascular access from the carotis—were performed after data collection from the right ventricle, which is a laborious procedure. In certain animals, by the time of left ventricular measurements the haemodynamic system became unstable, therefore they were excluded from the analysis. Furthermore, in some animals the quality of pressure recording was not sufficient to measure precise LVEDP or RVEDP (and Tau, mindP/dt, maxdP/dt) by the applied analysis module of the software.

Data was collected using SPR-671 1.4F catheters (Millar Instruments Inc., Houston, TX, USA) coupled to a Millar PCU-2000 pressure control unit and PowerLab 8/30 acquisition system (AD Instruments, Spechbach, Germany) with a sampling rate of 1 kHz. Before insertion into the vein, the catheter was placed into a small horizontal fluid-filled tube and calibrated to atmospheric pressure (accuracy 0.1 mmHg). Recordings were made over a 2-min period and analyzed with Powerlab Pro Software (AD Instruments, Spechbach, Germany). End-diastolic pressure (EDP), Tau index [Tau^*^Correspondent heart rate (Supplementary Figure S1)], mindP/dt and maxdP/dt were calculated from pressure tracings using a software analysis module.

### Assessment of right heart hypertrophy

Following haemodynamic measurements, animals were exsanguinated, lungs and hearts were perfused via the right ventricle with phosphate buffered saline. The heart was dissected free and kept on ice in PBS solution. After removing the atria from the heart, the right ventricle (RV) was dissected from the left ventricle plus septum (LV+S) and the separated regions were weighed to obtain the ratio of the right heart to the left ventricle plus septum [RV/(LV+S)] or to the animal's body weight (RV/BW).

### Pulmonary arterial banding

The pulmonary arterial banding (PAB) surgery was performed as previously described (Egemnazarov et al., 2015). Mice were anesthetized with intra-peritoneal fentanyl 0.05 mg/kg and midazolam 5 mg/kg and anesthesia was maintained with 2–3% isoflurane. After orotracheal intubation, the mice were mechanically ventilated using a volume controlled small animal ventilator MiniVent Type 845 (Hugosachs, March-Hugstetten, Germany). Ventilation settings were adjusted according to body mass using formulas (Tarnavski et al., 2004): Vt(ml) = 6.2 × M(kg)^1.01, where Vt(ml) is tidal volume and M(kg) is body mass of animal; and RR(min−1) = 53.5 × M(kg)^-0.26, where RR(min-1) is ventilation rate. A mouse weighing 25 g was ventilated with tidal volume of 150 μl and ventilation rate of 140 breaths/min.

An incision was made in the second left intercostal space using sterile technique, the pericardium was removed, and a partially occlusive titanium clip was placed around the main pulmonary artery (Weck, Research Triangle Park, NC, USA). The pulmonary artery was occluded to 0.3 mm, which approximately corresponds to 75% occlusion of the lumen diameter. The mice were then sutured and allowed to recover from anesthesia. Post-operative analgesic therapy was achieved by a subcutaneous injection of buprenorphine 0.1 mg/kg every 24 h for 3–5 days depending on animal's condition. Sham-treated animals underwent the same procedure with a vascular clip placed next to the vessel.

### Immunohistochemistry

Right lung was perfusion-fixed through the trachea with 4% formalin and embedded into paraffin blocks. The isolated heart was dissected in order to determine hypertrophy and processed also into paraffin for sectioning. Formalin-fixed paraffin-embedded lung and heart tissue was cut to 3,5 μm thick sections and stained with Masson's trichrome to determine collagen content. The level of fibrosis in right ventricle, in left ventricle + septum and around pulmonary vessels was quantified on the whole specimen area using image analysis software (Visiopharm, Hoersholm, Denmark). Antigen retrieval was performed with 0,01 M sodium citrate solution (pH 6.0) in 95°C. Duplicates were processed without primary antibodies as controls. ABCG2 protein expression was detected with anti-BCRP1/ABCG2 antibody (#AV43649, Sigma-Aldrich, Saint Louis, USA). Quantitative assessment of capillary density in the right heart and in left ventricle + septum was identified by anti-thrombomodulin (#AF3894, R&D systems, Minneapolis, USA) staining. The number of capillaries was reported relative to the total sample area in each case.

### Image analysis

Immunostained sections were digitized using slide scanner (Aperio, Oxford, UK) with an objective of 40× magnification. Digital image analysis was carried out by using Visiopharm Integrator System (VIS) semi-automated image analysis software, version 4.2.9.0. (Visiopharm, Hoersholm, Denmark).

### Cardiac fibroblast isolation

Mouse cardiac fibroblasts were isolated using a combined method of mechanical disruption and enzymatic digestion with collagenase buffer. Briefly, hearts from 9 weeks old (FVB/N) mice were removed and perfused with 1 ml of collagenase buffer (Krebs-Ringer buffer + 0.5 mg/ml collagenase II, 2.5 mM CaCl2 and 1 mg/ml BSA) in a retrograde manner from the aorta. The hearts were dissected free of connective tissue, vessels and atria. Right ventricles were carefully dissected, left ventricles were opened, and both were washed free of blood. 3-3 right- and left ventricles were combined and transferred into collagenase buffer where minced into small pieces. In between 3 rounds of digestion step (6 min/37°C), tissue pieces were triturated repeatedly with pipetting until no visible tissue fragments could be observed. Cell suspensions were collected and plated in T75 tissue culture flasks in DMEM/F-12 (1:1) (#21001-020, Gibco by life technologies, Paisley, UK) medium supplemented with 10% FCS, 1% L-glutamine and antibiotic-antimycotic solution. The medium was changed after 3 h, adherent cells were cultivated till ~90% confluency, when cells were detached using 0.25% (w/v) trypsin/EDTA, and recultured until passage 3 for experiments. The purity of the isolated cells was validated by an expression panel of characteristic fibroblast markers.

### Lung fibroblast isolation

Mouse lung fibroblasts were isolated using a mechanical disruption method. Briefly, lungs from 9 weeks old (FVB/N) mice were removed, dissected free of connective tissue, trachea and large vessels. After washing free of blood, lungs of 3 animals were combined, cut into small pieces and passed through a 100 μm mesh to avoid bigger vessels and bronchii. Cell suspensions were collected and plated in T75 tissue culture flasks in DMEM/F-12 (1:1) (#21001-020, Gibco by life technologies, Paisley, UK) medium supplemented with 10% FCS, 1% L-glutamine and antibiotic-antimycotic solution. The medium was changed after 2 days, adherent cells were cultivated till ~90% confluency, when cells were detached using 0.25% (w/v) trypsin/EDTA, and recultured until passage 3 for experiments. The purity of the isolated cells was validated by an expression panel of characteristic fibroblast markers.

### Cell culturing, cell stimulation

Human cardiac fibroblasts from the ventricle were obtained from Promocell. All experiments were performed with human cells in passage two to seven grown in complete fibroblast medium (Fibro Life® S2) or with mouse fibroblasts in passage 3 cultured in DMEM/F-12 (1:1) (10% FCS, 1% L-glutamine, 0.2% P/S). Prior to all stimulations, cells were kept in quiescent medium (VascuLife® Basal Medium 0%FCS with 1% P/S, or DMEM/F-12 (1:1) 0%FCS with 0.2% P/S for mouse fibroblasts) for 12 h, whereas treatments were performed in medium containing 2% FCS (VascuLife® Basal Medium 2% FCS with 1% P/S or DMEM/F-12 (1:1) 2%FCS with 0.2% P/S for mouse fibroblasts). A target-specific siRNA sequence, directed against human or mouse ABCG2 was purchased from Santa Cruz Biotechnology (#sc-41151 (h), #sc-37054 (m), Santa Cruz Biotechnology, Heidelberg, Germany). As control, a negative-control siRNA sequence was employed (#sc-37007, Santa Cruz Biotechnology, Heidelberg, Germany). Cells were transfected with siRNA using Amaxa Basic Nucleofector Kit (#VPI-1002, Lonza, Cologne, Germany). The siRNA-mediated down-regulation of the target protein was assessed 48 h after transfection by quantitative PCR and western blotting. Hypoxic treatment of the cells were carried out in an hypoxic work station from Biospherix (BioSpherix, Lacona, NY, USA) using fully-integrated incubators and work station with dynamic programmable hypoxic and non-condensing humidity control at 37°C. Hypoxic exposure (FiO2 of 0.01) was used for 48 h.

### Immunofluorescence

Mouse heart sections were deparaffinised at 60°C for 2 h, and incubated with 0.17% (w/v) trypsin for 20 min at 37°C, followed by blocking with 10% BSA in PBS for 40 min. Then samples were incubated overnight at 4°C with anti-ABCG2 (#AV43649, Sigma-Aldrich, 1:100 in 10% BSA) and anti-Vimentin (Abcam #ab11256, 1:200 in 10% BSA) antibodies, followed by incubation with Alexa Fluor 488 and 555-labeled antibodies for 45 min at room temperature (Life Technologies, 1:500 in 0.1% BSA). Sections were counterstained with 4′,6-diamidino-2-phenylindole dihydrochloride (DAPI) (Sigma-Aldrich) to identify nuclear DNA. For control staining duplicates were processed without primary antibodies.

Fibroblasts were grown on eight-well chamber slides and fixed by addition of 4% paraformaldehyde for 20 min at 37°C. Fixed cells were rinsed three times with PBS then permeabilized with 0.1% Triton-X100 in PBS for 15 min at room temperature followed by blocking with 3% BSA in PBS for 30 min. Then the cells were incubated overnight at 4°C with anti-ABCG2 (Abcam #ab3379, 1:100 in PBS), anti-Fibronectin (Abcam #ab23750, 1:100 in 0.1% BSA), anti-Vimentin (Abcam #ab11256, 1:400 in 0.1% BSA), anti-Periostin (Abcam #ab14041, 1:200 in 0.1% BSA), anti-S100A4 (Abcam #ab27957, 1:200 in 0.1% BSA) or anti-F-actin (Life technologies #A34055-conjugated, 1:80 in 0.1% BSA) antibodies respectively, followed by incubation with Alexa Fluor 555-labeled secondary antibodies (Life Technologies, 1:1,000 in PBS, except F-actin) for 30 min at room temperature. Cells were counterstained with DAPI (Sigma-Aldrich) to identify nuclear DNA. For control staining duplicates were processed without primary antibodies. Images were taken using Olympus Basic BX61VS Fluorescence microscope at magnification of ×40.

### Proliferation assay

Human cardiac fibroblasts were silenced as described above, later seeded in 96 well plates 20,000 cells/well. The following day media was changed to VascuLife® Basal Medium (2% FCS, 1% P/S) preincubated for 12 h in a hypoxic chamber, and the cells were placed in hypoxia (FiO2 of 0.01). After 24 h, preincubated VascuLife® Basal Medium (2% FCS, 1% P/S) was administered together with 2 μCurie/ml [3H]-thymidine (American Radiolabeled Chemicals, St.Louis, USA). Proliferation rate was determined by thymidine incorporation after 24 h as an index of DNA synthesis, measured as radioactivity by radioactive scintillation counter (Wallac 1450 MicroBetaTriLux, Minnesota, USA). Experiments were performed in quintuplicates.

### Hydroxyproline assay

Silenced human or mouse fibroblasts were seeded in 6 well plates, 200,000 cells/well. The following day, media was changed to VascuLife® Basal Medium (2% FCS, 1% P/S for human cells) or DMEM/F-12 (1:1) (2%FCS with 0.2% P/S for mouse fibroblasts) medium preincubated for 12 h in a hypoxic chamber, and cells -treated by this preconditioned media- were placed in hypoxia (FiO2 of 0.01) for 48 h. Later, cells were harvested and protein samples were hydrolysed in 6 M HCl at 95°C for 20 h. Following incubation, the tubes were cooled to room temperature and centrifuged for 10 min at 13,000 × g. Supernatants were×incubated with the detection reagents provided in the kit (Quickzyme Biosciences, Leiden, The Netherlands) at 60°C for 2 h and plate was read at 570 nm using spectrophotometer.

### Western blot analysis

Protein extracts of mouse ventricles or fibroblasts were prepared in RIPA buffer containing protease-inhibitor and phosphatase-inhibitor tablets (Roche, Vienna, Austria). After mechanical disruption of tissue samples, equivalent amounts of protein were separated on a SDS polyacrylamide gel, followed by electro-transfer to nitrocellulose membrane. Nonspecific antibody binding was blocked by incubation in 5% (m/v) non-fat dry milk powder in TBS-T (20 mM Tris-Cl, pH 7.5, 150 mMNaCl, 0.1% (v/v) Tween 20) at room temperature for 1 h. Then, samples were incubated overnight at 4°C with antibodies: anti-ABCG2 (#ab3380, Abcam) and anti-α-tubulin (#ab4074, Abcam) for ventricular tissues, and anti-ABCG2 (#AV43649, Sigma-Aldrich, Saint Louis, USA) and anti-GAPDH (#sc-25578, Santa Cruz Biotechnology, Heidelberg, Germany) for fibroblasts. After 1 h incubation in room temperature with peroxidase-labeled secondary antibody (Pierce Biotechnology, Rockford, USA), specific immunoreactive signals were detected using Enhanced ChemiLuminescence kit (Amersham Biosciences, Buckinghamshire, UK) or West Pico Substrate (Thermo Scientific, Rockford, USA).

### RNA extraction and quantitative real-time (Q-)PCR analysis

RNA was extracted from mouse formalin-fixed paraffin-embedded ventricle samples, using RNeasy FFPE Kit (#73504, Qiagen, Hilden, Germany). From cells RNA was extracted using spin-columns of peqGOLD Total RNA isolation kit (PeqLab, Erlangen, Germany). Nanodrop 2000c spectrophotometer (Nanodrop, Rockland, USA) was used to quantify the concentration and the purity of the isolated total RNA. Extracted RNA was reverse transcribed using iScript cDNA Synthesis Kit (BioRad, CA, USA). The final reaction mixture was incubated for 5 min at 25°C, followed by 30 min at 42°C and the reaction was terminated by heating at 85°C for 5 min. The reverse transcription reaction product was directly used in the PCR reaction or stored at −20°C. Gene expression was measured by quantitative real time PCR analysis using QuantiFast SYBR PCR Kit (Qiagen, Hilden, Germany). Reaction mixtures contained SYBR Green mastermix, forward plus reverse primers (Supplementary Table S1), and cDNA samples. Amplification was quantified with Lightcycler 480 system (Roche) using the next protocol: 1 × (95°C, 5 min); 40 × (95°C, 10 s; 60°C, 30 s). The data for amplification curves were acquired after the extension phase at 60°C. After amplification, melting curves were analyzed for validating PCR products. Genes were measured in duplicates. The expression of targeted genes are shown as ΔCT values normalized to the level of Beta-2 microglobulin (B2 mg) housekeeping gene using the averaged CT values by the formula ΔCT = CT(Housekeeping Gene)—CT(Gene of Interest).

### Cytokine and chemokine measurements

Circulating cytokine and chemokine profiling was performed on blood-derived serum samples from hypoxia-treated WT and ABCG2 KO mice (n:4 WT and 4 KO) using a dedicated mouse cytokine array kit (#ARY006, R&D systems, Minneapolis, USA). The array was performed according to manufacturer's protocol. Briefly, 50 μl serum samples were mixed with the provided biotinylated antibody cocktail, then incubated overnight (4°C) with the array nitrocellulose membrane containing duplicates of antibody spots against the chemokines. The chemiluminescent signal produced in the following step was proportional to the amount of captured cytokines. Developed X-ray films were analyzed using ImageJ software (Bethesda, Maryland, USA). Correspondent pixel densities are shown as arbitrary units normalized to the given reference spots of the membranes.

### Statistical analysis

Numerical values are given as means ± SD for all measurements. Statistical analysis was performed with SPSS Statistics software (IBM, USA: SPSS Inc.) and GraphPad Prism 5. Differences according to the genotype (KO vs. WT) and the treatment (normoxia vs. hypoxia) and their interactions (GxH) were analyzed by adjusted rank transformed two-way ANOVA (Leys and Schumann, 2010). Due to the exploratory character of the study, no *p*-value adjustment was applied for the analysis of main effects and interactions in the different parameters. For *post hoc* analysis of the interaction, KO and WT mice were compared separately in both treatment (normoxia vs. hypoxia), and normoxia vs. hypoxia were compared separately within both genotypes using Bonferroni adjusted Mann-Whitney *U* Test (four comparisons). For comparison of two groups, Mann-Whitney *U* test was carried out (*p* < 0.05 were considered significant). In the figures, only the results of the applied *post hoc* analysis or the Mann-Whitney *U* test are shown to provide information about intergroup contrasts. The general effect of hypoxia and genotype is presented in the adjusted rank transformed two-way ANOVA Supplementary Table S2.

## Results

### ABCG2 knockout has no influence on hypoxia-induced pulmonary hypertension

**Figure 1:**
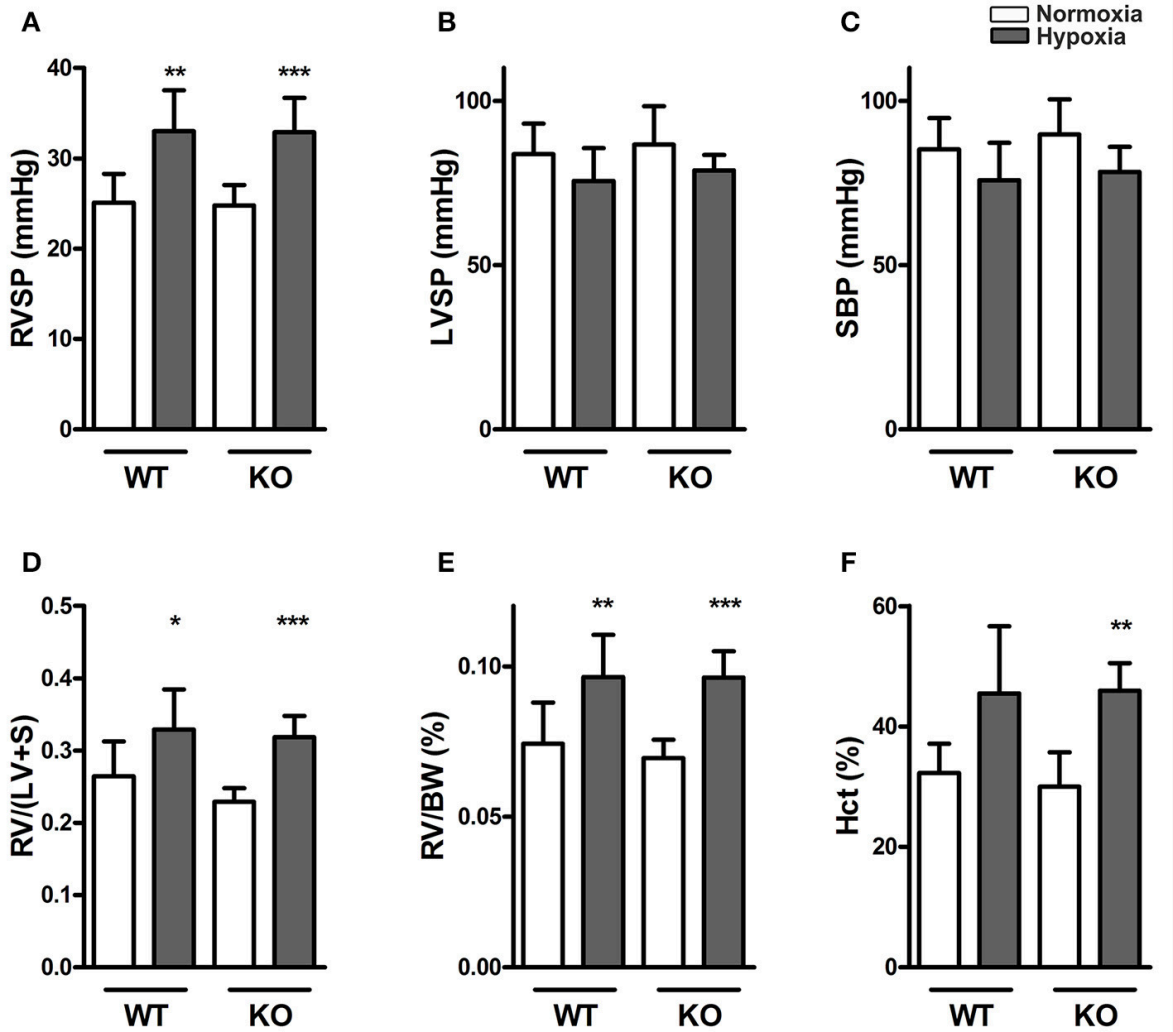
**Knocking out ABCG2 does not influence hypoxia-induced pulmonary hypertension**. Bar graphs summarize **(A)** right ventricular systolic pressure (RVSP) (*n* = WT nox:9, WT HOX:9, KO nox:10, KO HOX:11), **(B)** left ventricular systolic pressure (LVSP) (*n* = WT nox:8, WT HOX:8, KO nox:5, KO HOX:7) and **(C)** systolic blood pressure (SBP) (*n* = WT nox:9, WT HOX:9, KO nox:5, KO HOX:11) of WT and ABCG2 KO mice exposed to normoxia or chronic hypoxia. **(D)** Right ventricular hypertrophy shown by right ventricular (RV) to left ventricular plus septum (LV+S) ratio) (*n* = WT nox:9, WT HOX:10, KO nox:11, KO HOX:11) and **(E)** RV to body weight (BW) ratio (*n* = WT nox:9, WT HOX:10, KO nox:11, KO HOX:11). Percentage of blood haematocrit (Hct) levels in mice after normoxia or chronic hypoxia shown in subset **(F)** (*n* = WT nox:7, WT HOX:7, KO nox:7, KO HOX:7). Bar graphs represent values as means ± SD (^*^ corresponds to hypoxic treatment within genotype; ^*^p < 0.05, ^**^p < 0.01, ^***^p < 0.001).

In normoxia, RVSP, LVSP, systolic blood pressure, Fulton index [RV/(LV +S)] and hematocrit showed no differences between the two genotypes. RVSP, Fulton index and hematocrit increased in response to chronic hypoxia, independent of genotype, indicating that the development of hypoxic pulmonary hypertension was not affected by ABCG2. There were no interactions of hypoxia and genotype in any of these parameters (Figures 1A–F).

### Significant diastolic dysfunction in ABCG2 KO mice in response to hypoxia

**Figure 2:**
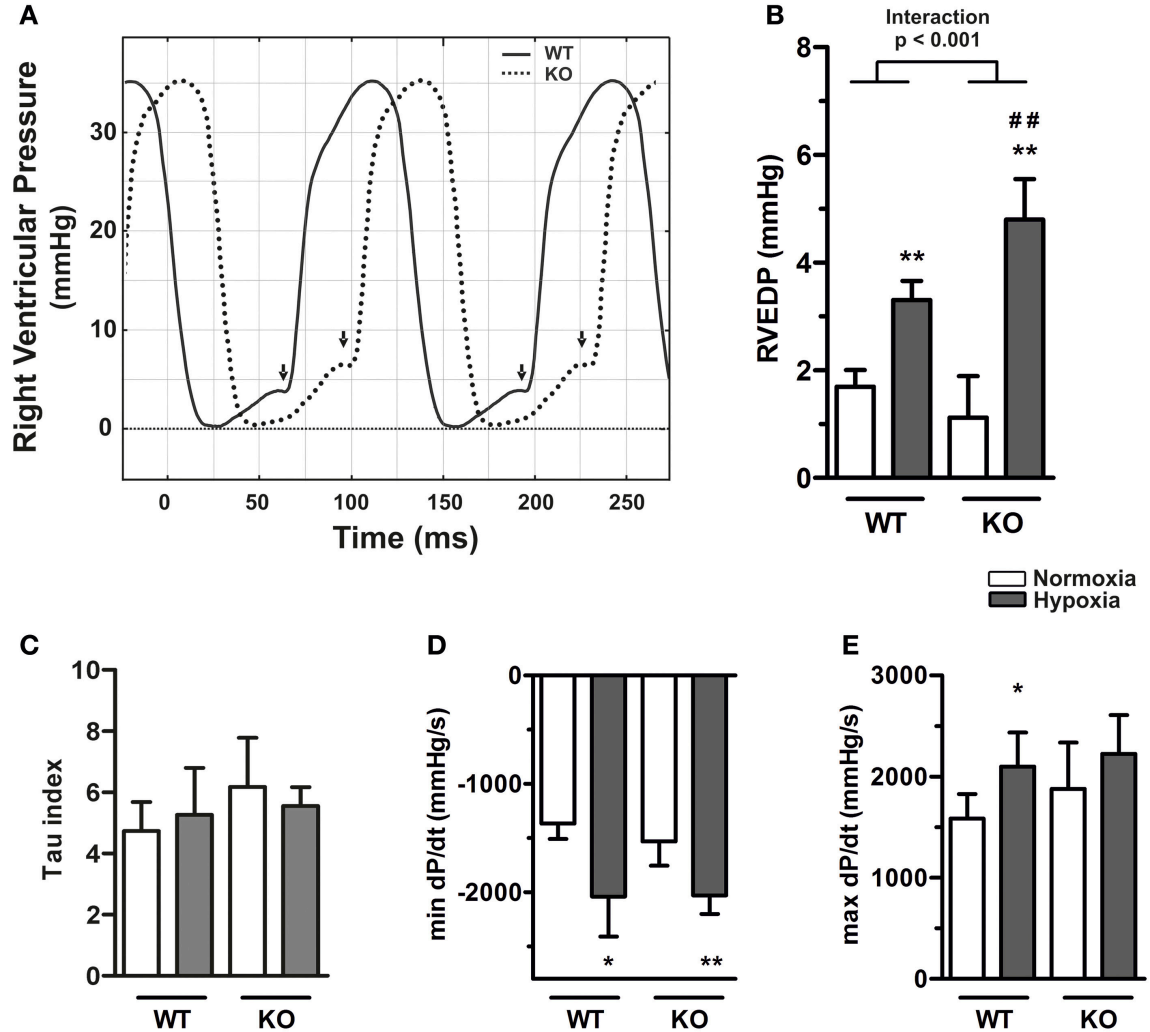
**Exacerbated right ventricular diastolic dysfunction in ABCG2 knockout mice in response to hypoxia. (A)** Representative pressure curves showing right ventricular pressure recordings, illustrating (arrows) end-diastolic pressure (RVEDP) alterations in ABCG2 KO (dashed line) compared to WT mice (continuous) under hypoxia. Summarized haemodynamic parameters showing **(B)** RVEDP (*n* = WT nox:7, WT HOX:7, KO nox:7, KO HOX:7), **(C)** Tau index (*n* = WT nox:6, WT HOX:7, KO nox:7, KO HOX:7), **(D)** mindP/dt (*n* = WT nox:7, WT HOX:7, KO nox:7, KO HOX:7) and **(E)** maxdP/dt (*n* = WT nox:7, WT HOX:7, KO nox:7, KO HOX:7) obtained from right ventricle of ABCG2 KO and WT mice exposed to normoxia or chronic hypoxia. Bar graphs represent values as means ± SD (Interaction p corresponds to hypoxia·genotype interaction, #corresponds to difference between genotypes in hypoxia, ^*^ corresponds to hypoxic treatment within genotype; ^*^p < 0.05, ^**^p ##*p* < 0.01).

Representative RV pressure recordings obtained from right heart catheterisation are shown from wild type (WT) and ABCG2 knockout (KO) mice under hypoxia in Figure 2A. Under normoxic conditions, the pressure of the right ventricle at the end of diastole (RVEDP) did not differ significantly between KO and WT mice (1.12 ± 0.29 mmHg vs. 1.69 ± 0.12 mmHg) (Figure 2B). Hypoxia caused a significant increase in RVEDP in both WT and KO mice, compared to the respective normoxic controls (*p* < 0.001, Supplementary Table S2). However, the effects were significantly stronger in KO than in WT mice as demonstrated by the interaction of the factors hypoxia and genotype (*p* for interaction < 0.001, Figure 2B). Thus hypoxia caused diastolic dysfunction in KO mice. The time constant of ventricular pressure decay [Tau (τ)] did not differ between any of the experimental groups (Figure 2C), indicating that the diastolic dysfunction was mainly relevant in the late diastolic phase. There was decreased mindP/dt in hypoxia independent of genotype (*p* < 0.001, Figure 2D). This is consistent with hypoxic pulmonary vasoconstriction causing larger pressure decay during early diastole. Similarly an increase was observed with maxdP/dt (Figure 2E, Supplementary Table S2).

**Figure 3:**
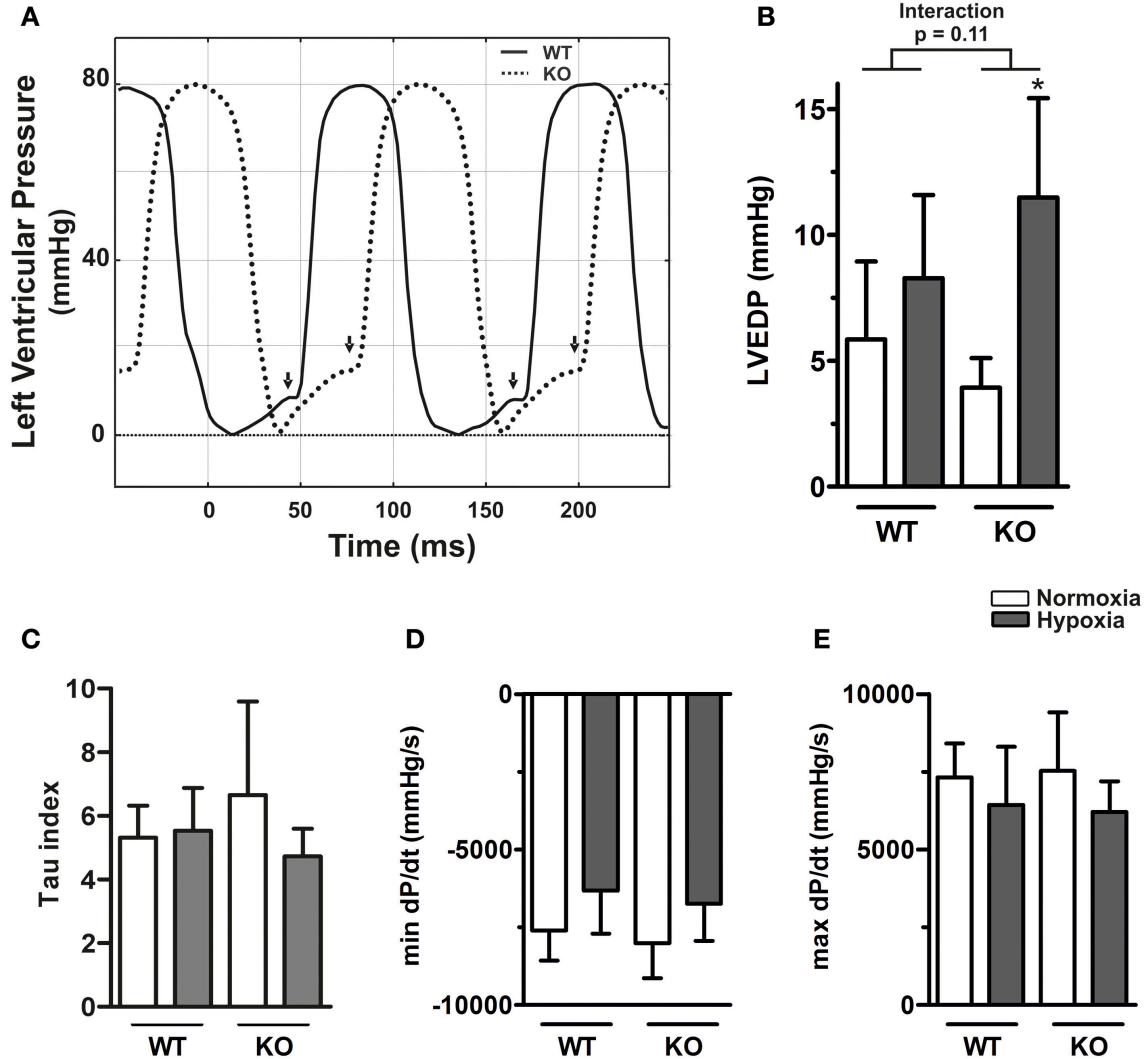
**Left ventricular diastolic dysfunction in ABCG2 knockout mice in response to hypoxia. (A)** Representative pressure curves showing left ventricular pressure recordings, illustrating (arrows) end-diastolic pressure (LVEDP) alterations in ABCG2 KO (dashed line) compared to WT mice (continuous) under hypoxia. Summarized haemodynamic parameters showing **(B)** LVEDP, **(C)** Tau index, **(D)** mindP/dt, and **(E)** maxdP/dt obtained from left ventricle of ABCG2 KO and WT mice exposed to normoxia or chronic hypoxia. (*n* = WT nox:5, WT HOX:7, KO nox:5, KO HOX:6) Bar graphs represent values as means ± SD (^*^corresponds to hypoxic treatment within genotype; ^*^p < 0.05).

Left ventricular end-diastolic pressure (LVEDP) of WT and KO mice did not differ in normoxia but hypoxia caused an elevation (*p* = 0.001, Supplementary Table S2). Although the effect was more pronounced in KO mice, the interaction between hypoxia and genotype did not reach significance (*p* for interaction = 0.11, Figure 3B), which might be due to lower sample sizes in these groups. In the left ventricle hypoxia had a significant effect on mindP/dt independent of genotype (*p* = 0.013, Figure 3D). However, neither the genotype nor hypoxia had any significant effects on Tau or maxdP/dt (Figures 3C,E, Supplementary Table S2).

### Increased ventricular fibrosis in ABCG2 KO mice under hypoxia, without changes in capillary density

**Figure 4:**
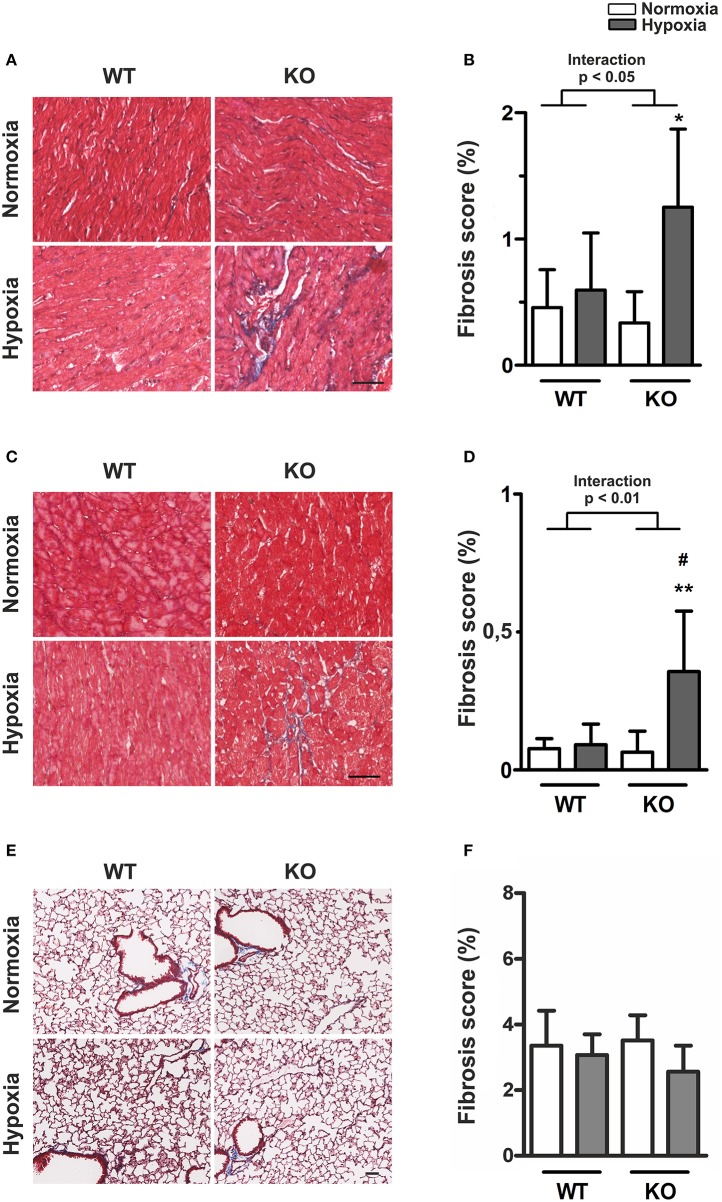
**Increased ventricular fibrosis in ABCG2 KO mice under hypoxia. (A)** Masson's trichrome staining shows presence of collagen (blue) in right ventricular sections of ABCG2 KO and WT mice after normoxic or hypoxic exposure. The same is shown for the left ventricle **(C)**, and in lung tissue in subset **(E)**. Quantification shows presence of collagen in the right- **(B)** and left ventricle **(D)**, as well as in the lung **(F)**. (*n* = WT nox:6, WT HOX:7, KO nox:7, KO HOX:7) Scale bar indicates 50 μm. Bar graphs represent values as means ± SD (Interaction p corresponds to hypoxia·genotype interaction, #corresponds to difference between genotypes in hypoxia, ^*^ corresponds to hypoxic treatment within genotype; ^#^*p* < 0.05, ^*^p < 0.05, ^**^p < 0.01).

Under normoxia, there were no differences in the RV (Figures 4A,B) and LV (Figures 4C,D) fibrosis scores of KO mice compared to WT. Hypoxia caused a significant increase in ventricular fibrosis scores in KO, but not in WT mice. This was true for the right (*p* for interaction < 0.05) and for the left ventricle (*p* for interaction < 0.01). Figure 4 shows that fibrosis was more pronounced in the RV but the response pattern in terms of hypoxia and genotype was the same in both ventricles. The observed increase in RV fibrosis correlated significantly with RVEDP (*p* < 0.05, *R*^2^ = 0.59, see Supplementary Figure S2). On the other hand, neither hypoxia, nor the lack of ABCG2 had any significant effect on lung fibrosis (Figures 4E,F).

Previous studies in the transverse aortic constriction model showed that mice lacking ABCG2 have reduced angiogenesis in the LV, which was associated with cardiac hypertrophy (Higashikuni et al., 2010, 2012). To investigate the involvement of ABCG2 on the capillary density, we stained for thrombomodulin to visualize capillaries in RV and LV sections (see Supplementary Figure S3). Interestingly, neither hypoxia, nor the lack of ABCG2 had any effect on ventricular capillary density (see Supplementary Figures S3B,C, Supplementary Table S2). This indicates that the observed EDP elevation and ventricular fibrosis may occur completely independent of vessel density.

### Fibrosis-related genes in ventricular tissue of hypoxic mice

The expression profile of fibrosis-related molecular markers in the RV and LV was investigated by Q-PCR, in order to investigate ongoing fibrotic processes (see Supplementary Figure S4). At the end of 4 weeks hypoxic treatment, when myocardial fibrosis was already established, there were no significant changes in most of the investigated genes. In the RV, Col3A1 was significantly upregulated in ABCG2 KO mice in hypoxia (*p* < 0.05). This could be due to both, hypoxia or increased RV afterload, however, as there was no such increase in the LV, this indicates that the ongoing upregulation was mainly due to the persistently increased afterload in the case of the RV.

### Increased extracellular matrix production in ABCG2 silenced mouse isolated cardiac fibroblasts under hypoxia

**Figure 5:**
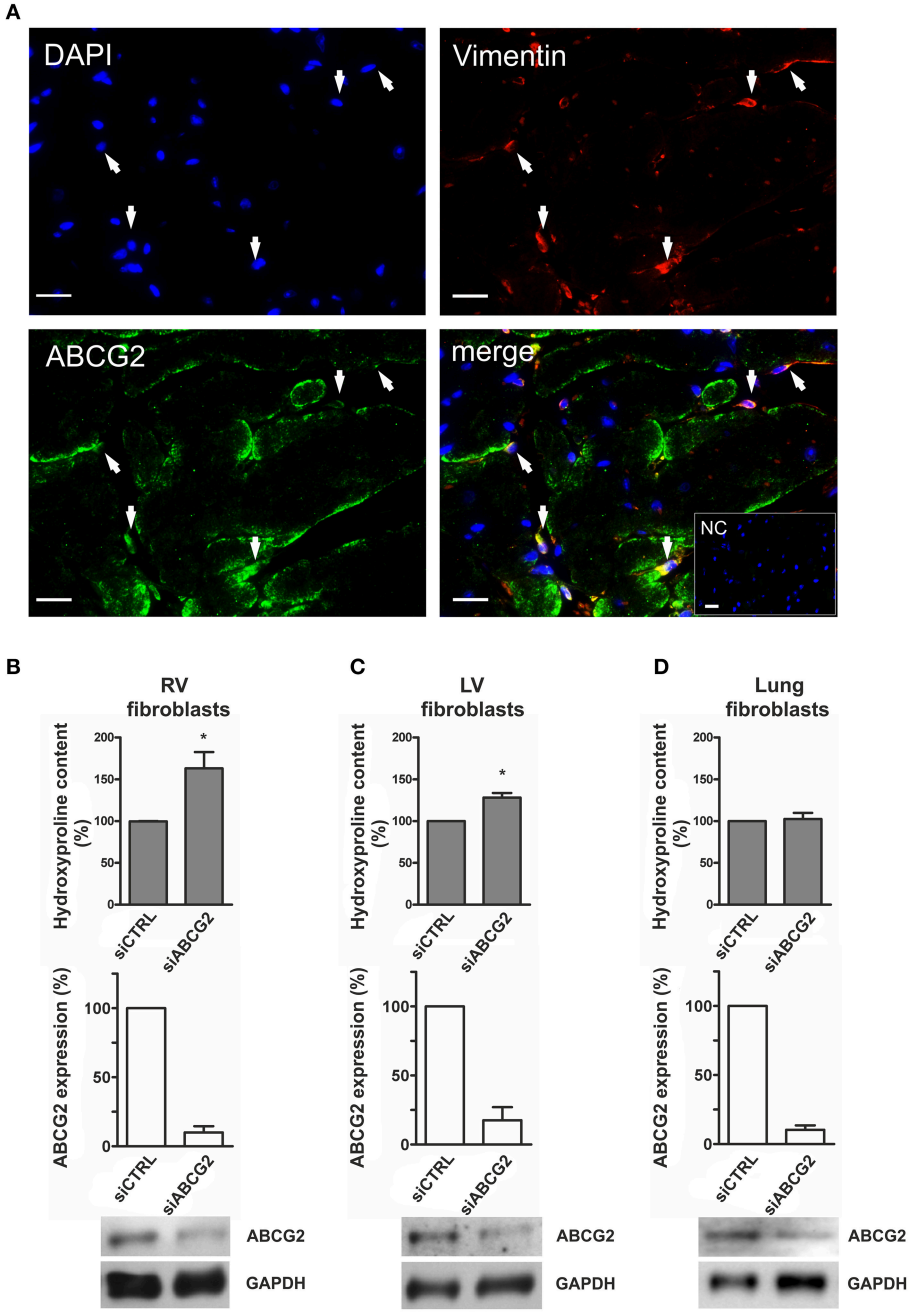
**Increased extracellular matrix production in ABCG2 silenced mouse isolated cardiac fibroblasts under hypoxia. (A)** Fluorescent immunostainings indicate DAPI nuclear staining (blue), vimentin (red), ABCG2 (green) and an overlaid picture (merge) in a single–plane image of the mouse ventricle. Arrows pointing out cardiac cells coexpressing ABCG2 and vimentin. Inset represents correspondent negative control. Scale bar = 20 μm. Graphs represent collagen content as percentage in mouse primary fibroblasts derived from right ventricles **(B)**, left ventricles **(C)** or from lungs **(D)** (*n* = 4), and show ABCG2 mRNA expression in correspondent fibroblasts after transfection with control siRNA or siABCG2 (*n* = 3). Western blot gel pictures represent protein levels of ABCG2 in the respective cells. Equalized loading was confirmed with GAPDH. Bar graphs represent values as means ± SD (^*^p < 0.05).

As shown in Figure 5A, ABCG2 was coexpressed with vimentin—fibroblastic marker in mouse ventricular tissue. Cardiac fibroblasts (see Supplementary Figure S5) derived from mouse right- (Figure 5B) and left ventricles (Figure 5C) showed increased collagen production in the absence of ABCG2 under hypoxia (*p* < 0.05). On the other hand, silencing of ABCG2 did not affect the extracellular matrix production of mouse primary lung fibroblasts in hypoxia (Figure 5D). These results suggest that in hypoxic mouse ventricular fibroblasts, -but not in pulmonary fibroblasts- ABCG2 protects against excess collagen production.

### Increased extracellular matrix production in ABCG2 silenced human primary cardiac fibroblasts under hypoxia

**Figure 6:**
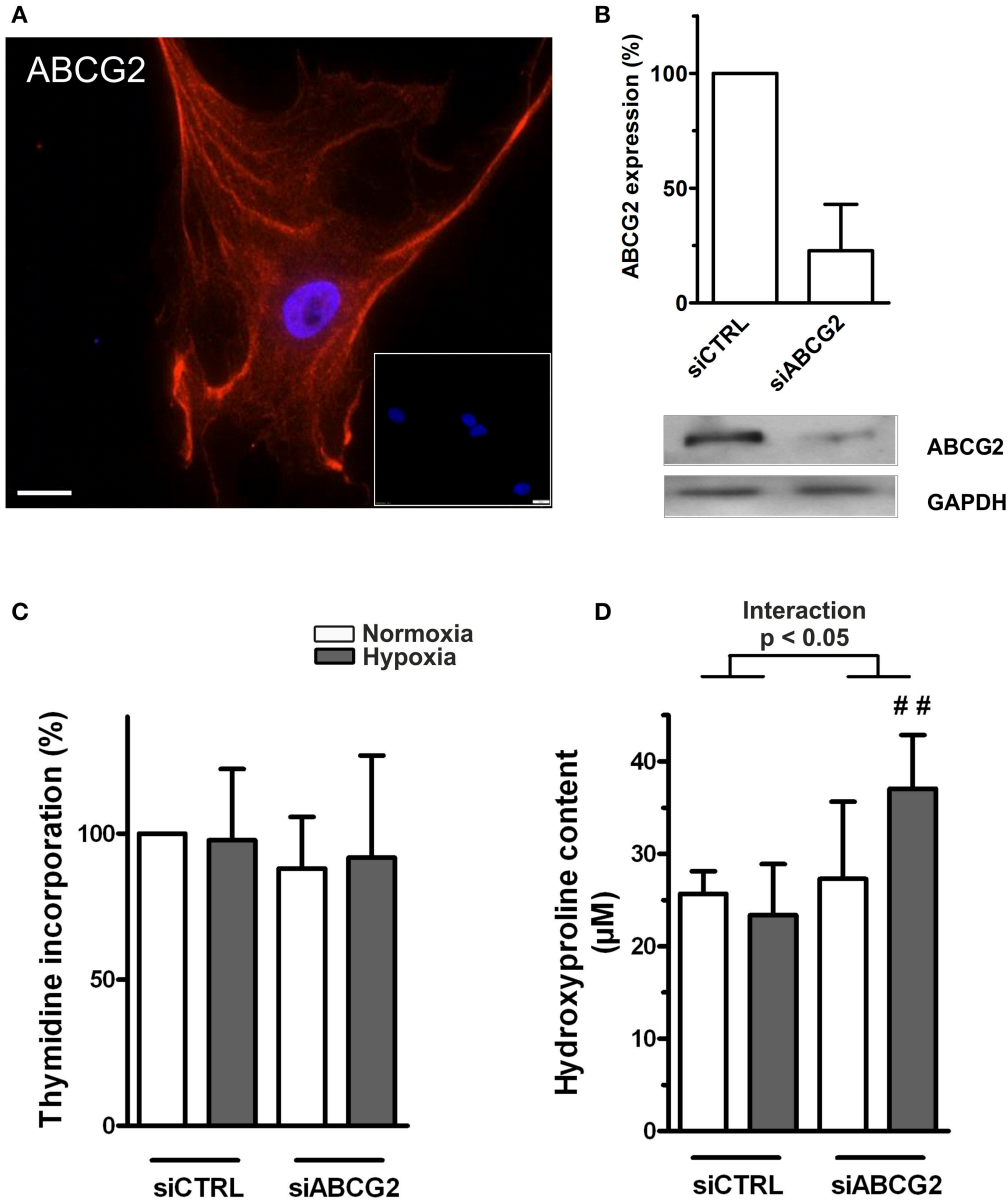
**Increased extracellular matrix production in ABCG2 silenced human primary cardiac fibroblasts under hypoxia. (A)** Fluorescent immunostaining indicate DAPI nuclear staining (blue) and ABCG2 (red) in a single–plane image of human cardiac fibroblast. Inset represents correspondent negative control. Scale bar = 20 μm. **(B)** Bar graph shows ABCG2 mRNA expression in human primary cardiac fibroblasts after transfection with control siRNA or siABCG2 (*n* = 3). Western blot gel picture represents protein levels of ABCG2 in human primary cardiac fibroblasts. Equalized loading was confirmed with GAPDH. **(C)** Human primary cardiac fibroblast proliferation, measured by incorporation of [3H]thymidine (*n* = 4). **(D)** Graph represents collagen content in human primary cardiac fibroblasts, measured by hydroxyproline assay (*n* = 5). Bar graphs represent values as means ± SD (Interaction p corresponds to hypoxia·genotype interaction, #corresponds to difference between genotypes in hypoxia, ^##^*p* < 0.01).

In human primary cardiac fibroblasts, the readouts were proliferation and the collagen production. Human cardiac fibroblasts expressed ABCG2 protein (Figures 6A,B). The efficiency of our siRNA against ABCG2 was quantified on the mRNA and protein level, and showed nearly 80% silencing (Figure 6B). Silencing of ABCG2 did not change primary cardiac fibroblast proliferation under normoxia or hypoxia (Figure 6C). However, when ABCG2 was supressed in human cardiac fibroblasts there was an increased collagen production under hypoxia showing significant interaction between ABCG2 silencing and treatment (*p* for interaction < 0.05, Figure 6D). This indicates that in human primary cardiac fibroblasts, ABCG2 plays a protective role against hypoxia-induced collagen production.

### Regulation of ABCG2 in the stressed mouse ventricle

**Figure 7:**
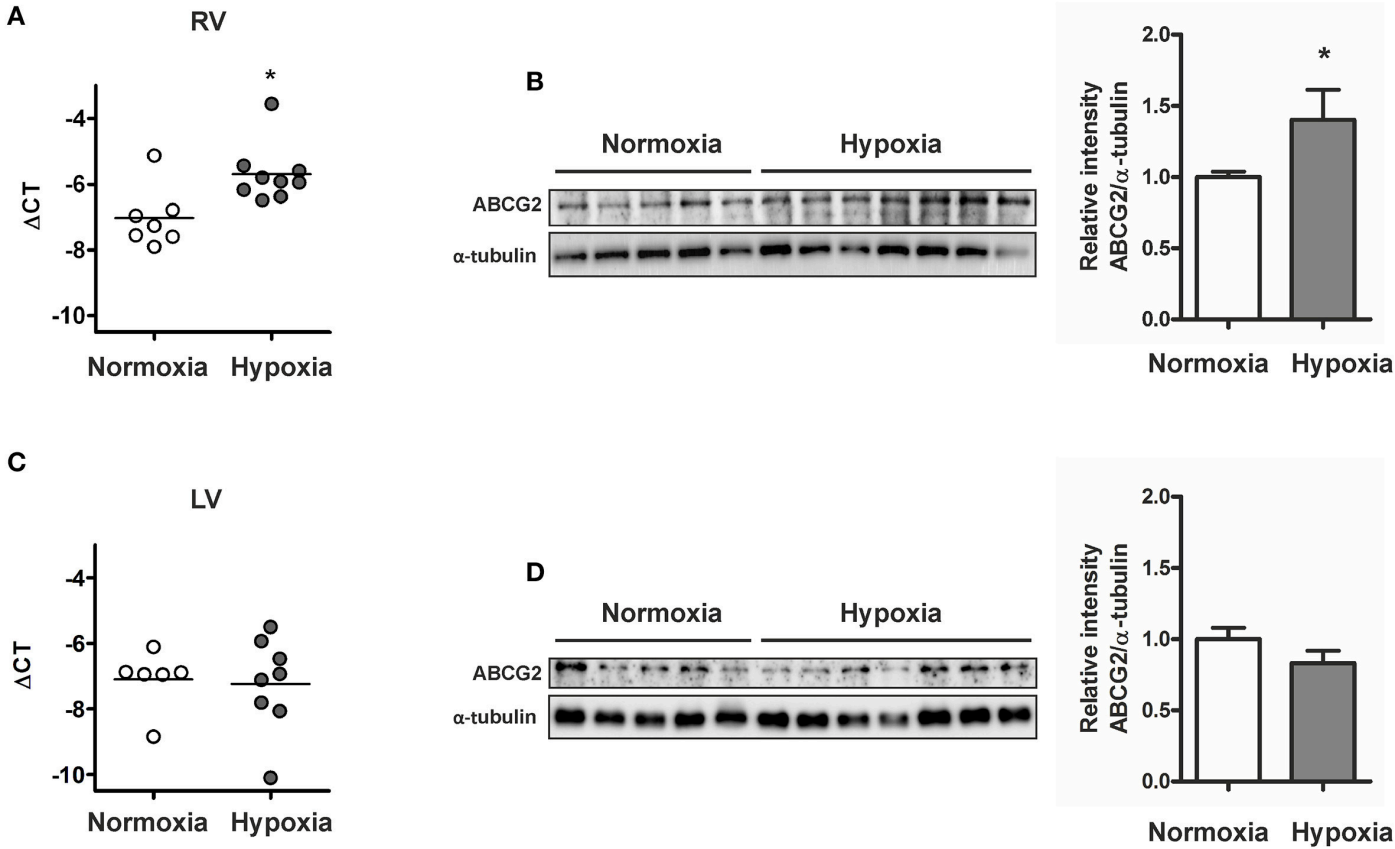
**Increased ABCG2 expression in the stressed right ventricle of chronic hypoxic mice**. Graph shows ABCG2 gene expression in right **(A)** - and left ventricles **(C)** of mice exposed to chronic hypoxia (*n* ≥ 6 for each group). Subset **(B,D)** shows western blot analysis of ABCG2 in right- and left ventricle homogenates respectively from mice after exposure to hypoxia for 4 weeks (*n* = 5 for normoxia, *n* = 7 for hypoxia). Signal densities were quantified using densitometric analysis. (^*^p < 0.05).

ABCG2 expression was significantly increased in the RV of chronic hypoxia-treated mice (*p* < 0.05, Figures 7A,B) but not in the hypoxic left ventricle (Figures 7C,D). Upregulation of the ABCG2 gene was observed in RVs of pulmonary artery banded mice (*p* < 0.01, see Supplementary Figure S6), suggesting that after chronic hypoxic exposure, afterload but not hypoxia regulates ABCG2 in the myocardium.

## Discussion

The present study investigated the role of the ABC membrane transporter family member ABCG2 in hypoxia on cardiac and pulmonary vascular function. Our results show, that ABCG2 knockout mice developed significant biventricular diastolic dysfunction and fibrosis under chronic hypoxia. In contrast, ABCG2 KO animals showed lung vascular remodeling, increased pulmonary pressure and RV hypertrophy similar to WT mice, thus the absence of ABCG2 had no influence on the development of hypoxia-induced PH. These results may help in understanding the protective role of ABCG2 for function and structure of the heart under hypoxic conditions and raise some concern against the use of specific inhibitors for cancer therapy.

Exposure of mice to chronic hypoxia causes many of the anatomic and haemodynamic alterations of the lung and the heart that are known from patients with chronic lung disease (Stenmark et al., 2009; Ryan et al., 2011), thus recapitulates the human chronic hypoxemic conditions. After development of pulmonary arterial remodeling, the RV has to cope with an increased afterload which initially results in the elevation of systolic and diastolic ventricular pressures, leading to RV hypertrophy and finally to RV decompensation (Leite-Moreira et al., 1999; Humbert et al., 2004; Martos et al., 2007; Vonk Noordegraaf and Galie, 2011; van de Veerdonk et al., 2011; Paulin et al., 2015). We found significantly increased ABCG2 levels in the right- but not the left ventricles of hypoxic animals. In chronic hypoxic pulmonary hypertensive mice the RV had to adapt to persistently increased afterload in addition to hypoxia. This might be the reason why gene expression differences could be detected only in the RV at the end of the hypoxic exposure, indicating a putative role of this transporter in the stressed heart. This notion is also supported by an increased ABCG2 expression in the RV of pulmonary arterial banded (PAB) mice (see Supplementary Figure S6), an animal model recapitulating solely RV pressure overload observed in pulmonary hypertension.

Incomplete ventricular relaxation at end-diastole causes elevated filling pressure which is the main characteristic of diastolic dysfunction. In the LV, elevated ventricular EDP is a well-established indicator of heart failure and predictor of mortality (Salem et al., 2006). When ABCG2 KO mice were exposed to chronic hypoxia, we observed overt worsening of biventricular diastolic function, as indicated by a significant increase in EDP. Parameters describing early diastolic relaxation, such as Tau or mindP/dt remained unchanged between genotypes, collectively indicating a disturbed ventricular function exclusively at the end of diastole. Such diastolic changes can be caused by myocardial fibrosis, which may lead to functional changes of the ventricle (Rain et al., 2013). Extracellular matrix deposition may provide a cardioprotective effect by increasing myocardial structural resistance, protecting against elevated pressure (Krenning et al., 2010). However, excess fibrosis can decrease ventricular performance (Chiao et al., 2012).

Elevated myocardial fibrosis is one of the main pathological features associated with diastolic heart failure (Martos et al., 2007; Rain et al., 2013). Furthermore, therapies ameliorating ventricular fibrosis have been shown to improve diastolic function (Brilla et al., 2000; Diez et al., 2002). Our study shows that the hypoxia-induced structural changes in the ventricular myocardium are markedly exaggerated in ABCG2 KO mice, suggesting that ABCG2 protects from hypoxia-induced myocardial fibrosis. Interestingly, the same response pattern in terms of diastolic dysfunction was found in the right and left ventricle. This suggests that hypoxia *per se* and not hypoxia-induced changes in afterload were the main cause for elevated ventricular end-diastolic pressure and myocardial fibrosis in ABCG2 KO animals.

In comparison to the WT animals ABCG2 KO mice has elevated circulating levels of G-CSF, IL-1ra, CXCL1, and MCP-1 in hypoxia (see Supplementary Figure S7). The elevation of these fibrosis-associated cytokines (Sugano et al., 2005; Dobaczewski and Frangogiannis, 2009; Vicenova et al., 2009; Sahin and Wasmuth, 2013) might have also contributed to the increased ventricular fibrosis, observed in ABCG2 KO mice under hypoxia. Fibroblast-mediated matrix production may lead to increased ventricular fibrosis which may result in elevated end-diastolic pressure, as it can be also implied by a correlation of RVEDP with right ventricular fibrosis. It is well accepted that the excess fibrous tissue deposition in the heart is generally due to increased extracellular matrix synthesis and/or proliferative potential of cardiac fibroblasts (Camelliti et al., 2005). Herein we also showed that cardiac fibroblasts do express ABCG2 transporter in the mouse heart. When these primary cells were isolated from the ventricular tissue, they showed an increased collagen production upon ABCG2 silencing under hypoxia. Nevertheless, fibroblasts from mouse lungs did not show these changes, which was in agreement with our *in vivo* observations with increased ventricular fibrosis in KO mice under hypoxia.

In line with the observations in the mouse ventricular fibroblasts, we showed that human primary cardiac fibroblasts do express ABCG2 and that its silencing significantly increased their collagen production under hypoxia. This indicates that the results obtained from the mouse model might be relevant for cardiac fibrosis and diastolic dysfunction in humans. It has been shown that ABCG2 provides cytoprotective effects under oxidative stress, a condition very often associated to hypoxia (Maher et al., 2014). Another elegant study suggested that ABCG2 protects from hypoxia by preventing the accumulation of porphyrins, explaining why the lack of ABCG2 increases the sensitivity to hypoxia (Krishnamurthy et al., 2004). It is likely that in the hypoxic heart, the lack of ABCG2 leads to an accumulation of metabolic products, directly triggering collagen production in cardiac fibroblasts.

In spite of these findings, the development of ABCG2 inhibitors is being expedited, because ABCG2 provides multidrug resistance for tumor cells by active drug efflux, thus the bioavailability of anticancer drugs can be improved by co-administration with ABCG2 inhibitors (Breedveld et al., 2006; Wu et al., 2008). Therefore, close attention to the cardiac side effects of this new anticancer strategy is warranted, especially in hypoxemic patients (Kessler et al., 1996; Nieto et al., 2012; Campos-Rodriguez et al., 2013; Laveneziana et al., 2013) and those with ischemic heart disease. The present findings suggest, that ABCG2 inhibition might cause adverse cardiac effects, if it were to be used in cancer patients.

## Conclusions

We provide evidence that loss of ABCG2 under hypoxia leads to biventricular fibrosis and diastolic dysfunction, but does not affect the development of pulmonary hypertension. In addition, targeting ABCG2 by siRNA in human primary cardiac fibroblasts increased their collagen production under hypoxia. Collectively, we have presented both *in vivo* and *in vitro* findings strongly suggesting ABCG2 as an important player for diastolic dysfunction under hypoxia. The use of ABCG2 inhibitors in cancer patients therefore might cause drug-induced cardiotoxicity, particularly in hypoxemic patients or ischemic heart disease.

## Author contributions

Conception, hypothesis delineation and study design: BMN, AO, and HO. Data acquisition, analysis and interpretation: BMN, CN, BE, and AA. Drafting the manuscript for important intellectual content: BMN, AO, HO, GK, and RS.

## Disclosures

AO reports grants from Pfizer Inc., during the conduct of the study; HOreports grants from Bayer, Unither Pharmaceuticals, Actelion Pharmaceuticals Ltd. and Pfizer Inc., personal fees from Bayer, Unither Pharmaceuticals, Actelion Pharmaceuticals Gilead Sciences, Inc., Encysive Pharmaceuticals Ltd. GlaxoSmithKline and Nebu-Tec, personal fees and non-financial support from Bayer, Unither Pharmaceuticals, Actelion Pharmaceuticals Ltd., Pfizer Inc., Eli Lilly and GK, outside the submitted work.

### Conflict of interest statement

The authors declare that the research was conducted in the absence of any commercial or financial relationships that could be construed as a potential conflict of interest.
