# Supplementary material for: Lack of ABCG2 Leads to Biventricular Dysfunction and Remodeling in Response to Hypoxia
Source: Front Physiol. 2017 Feb 21;8:98. doi: 10.3389/fphys.2017.00098 (PMC5318436; doi:10.3389/fphys.2017.00098)
Supplement: Supplementary file 1 [file DataSheet1.DOC]

**ONLINE SUPPLEMENTARY DATA**

**Lack of ABCG2 leads to biventricular dysfunction and remodelling in response to hypoxia**

Bence M. Nagy, Chandran Nagaraj, Bakytbek Egemnazarov, Grazyna Kwapiszewska, Rudolf E. Stauber, Alexander Avian, Horst Olschewski*, Andrea Olschewski

**Supplemental Figure legends**

**Figure S1. Heart rates acquired during haemodynamic measurements**

Bar graphs represent values as means ±SD.

**Figure S2.** **Correlation of RVEDP with right ventricular fibrosis in hypoxic mice**

**Figure S3. Comparable ventricular capillary density in WT and ABCG2 KO mice**

Capillaries are visualized by thrombomodulin staining in ABCG2 KO and WT right (A) and left ventricles (C) of mice exposed to normoxia or chronic hypoxia. Quantification of capillary density in the RV and LV are shown in subset B and D respectively (n= WT nox:7, WT HOX:7, KO nox:7, KO HOX:6).

**Figure S4. Fibrosis-related genes in mouse ventricular tissue after 4 weeks of hypoxia**

Graphs show Collagen1A1, Collagen3A1, Fibronectin and TIMP1 mRNA expression in the (A) right and (B) left ventricles of WT and KO mice after exposure of 4 weeks chronic hypoxia. (n≥6 for each group, * corresponds to hypoxic treatment, *p<0.05).

**Figure S5.Characterisation of isolated mouse fibroblasts**

Immunofluorescence images display positive staining for characteristic markers (periostin, S100A4, vimentin, fibronectin) in fibroblasts isolated from mouse ventricle (A) and lung (B) respectively. NC corresponds to negative control. Scale bar= 50 µm.

**Figure S6.Regulation of ABCG2 in PAB right ventricles**

Graph shows right ventricular systolic pressure (A) and right ventricular hypertrophy (B) shown by right ventricular (RV) to left ventricular plus septum (LV+S) ratio in sham operated and pulmonary artery banded mice (n= Sham:8, PAB:9). (C) ABCG2 gene expression in right ventricles of mice underwent pulmonary artery banding. Bar graphs represent values as means ±SD (**p <0.01, ***p <0.001).

**Figure S7.Circulating cytokine/chemokine profiling of hypoxic mice**

Representative pictures show nitrocellulose-based mouse cytokine/chemokine array membranes performed on serum samples from hypoxia-treated WT and ABCG2 KO mice (A). (B) Correspondent pixel densities normalized to the given reference spots of the membranes are shown as arbitrary unit ± SEM. (n= 4).

**Supplemental Figures**

**
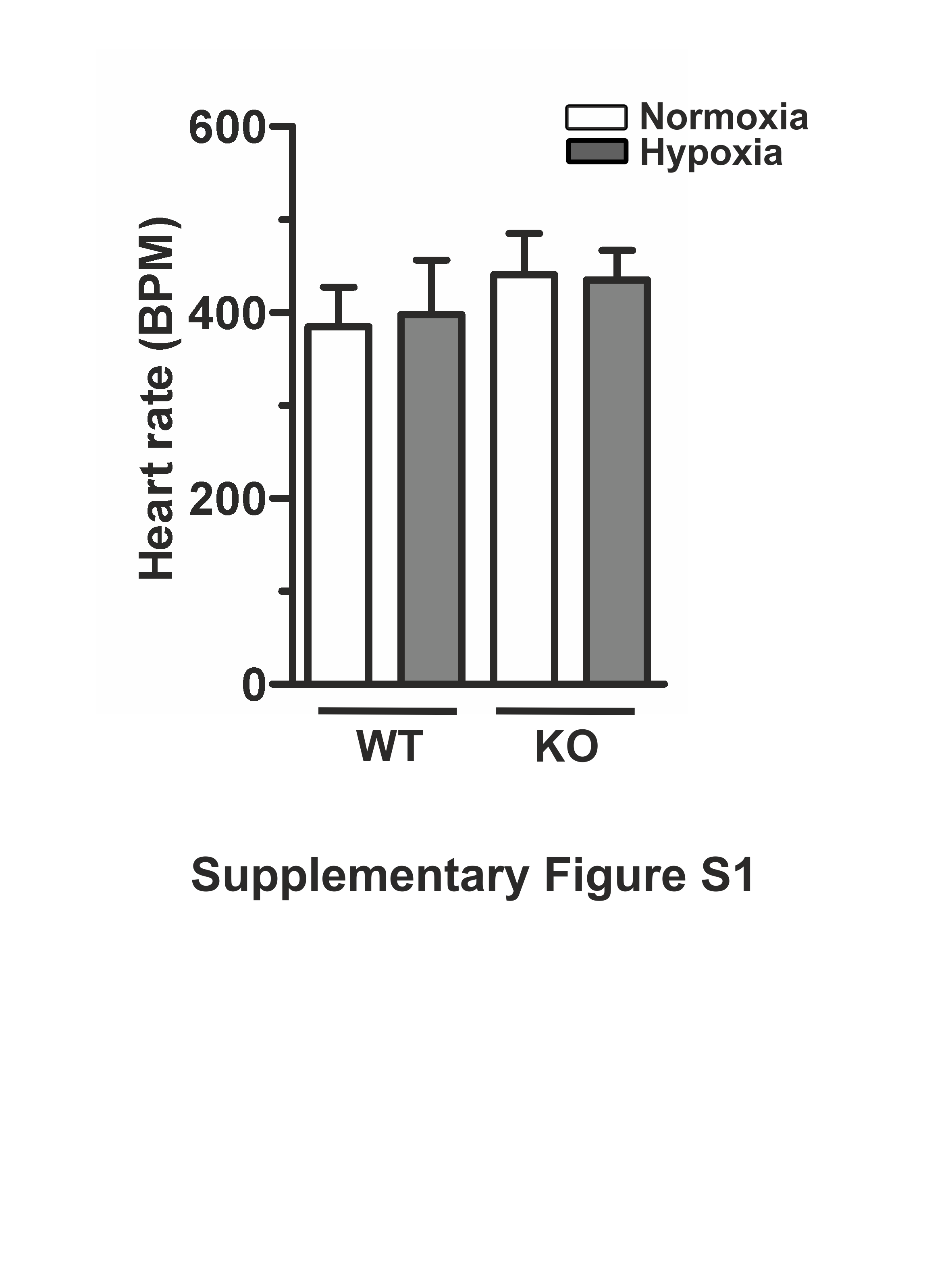
**

**
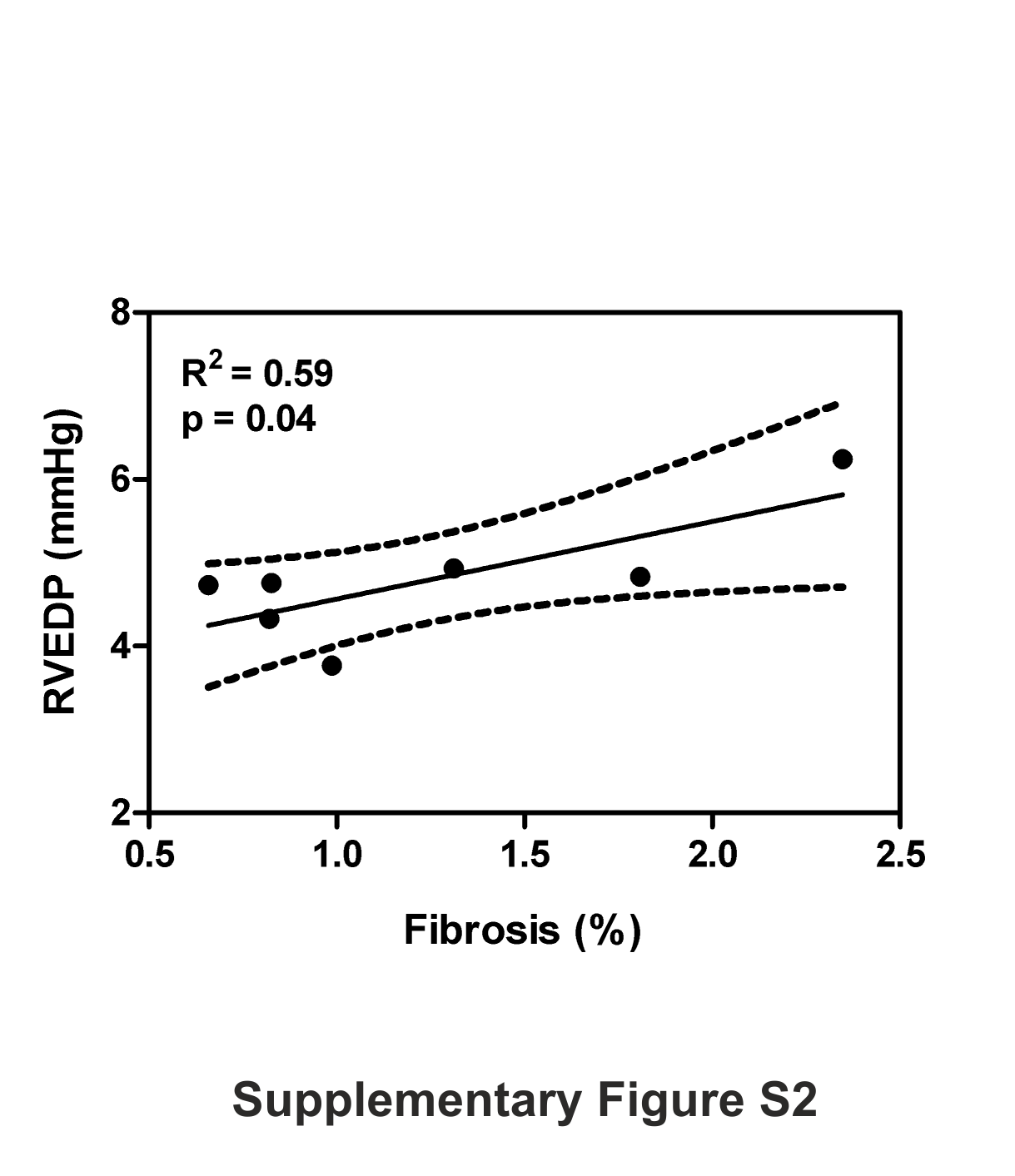
**

**
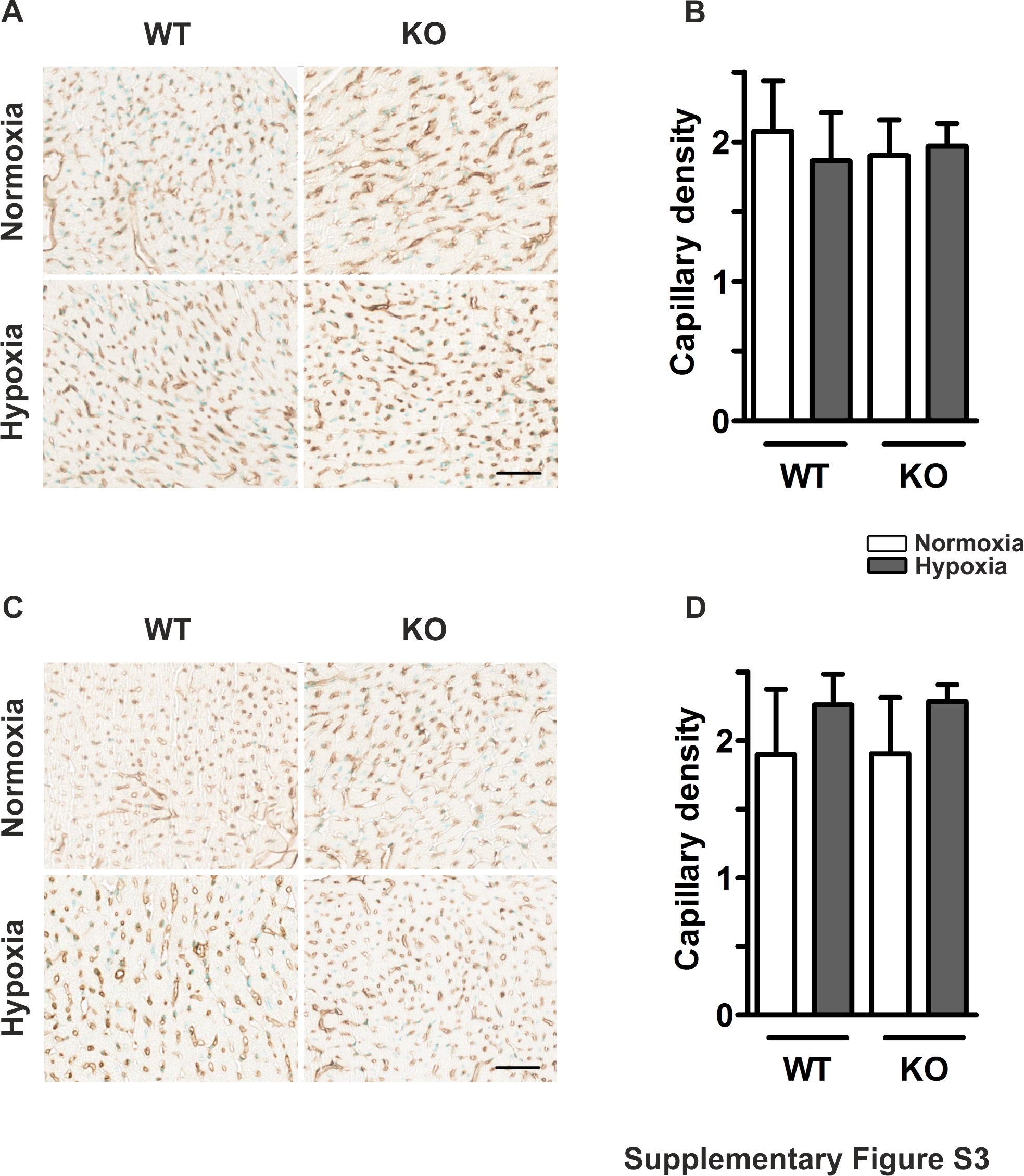
**

**
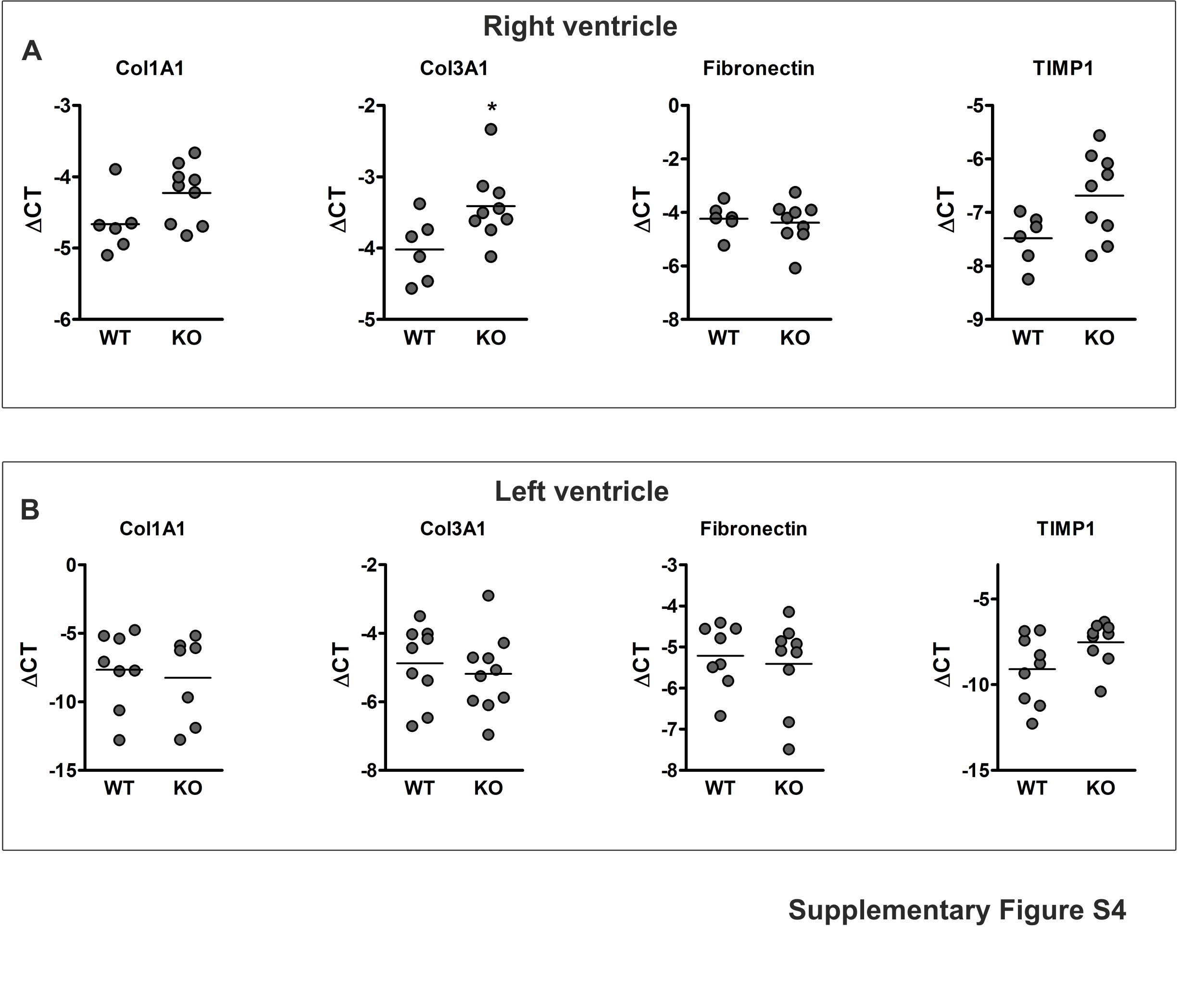
**

**
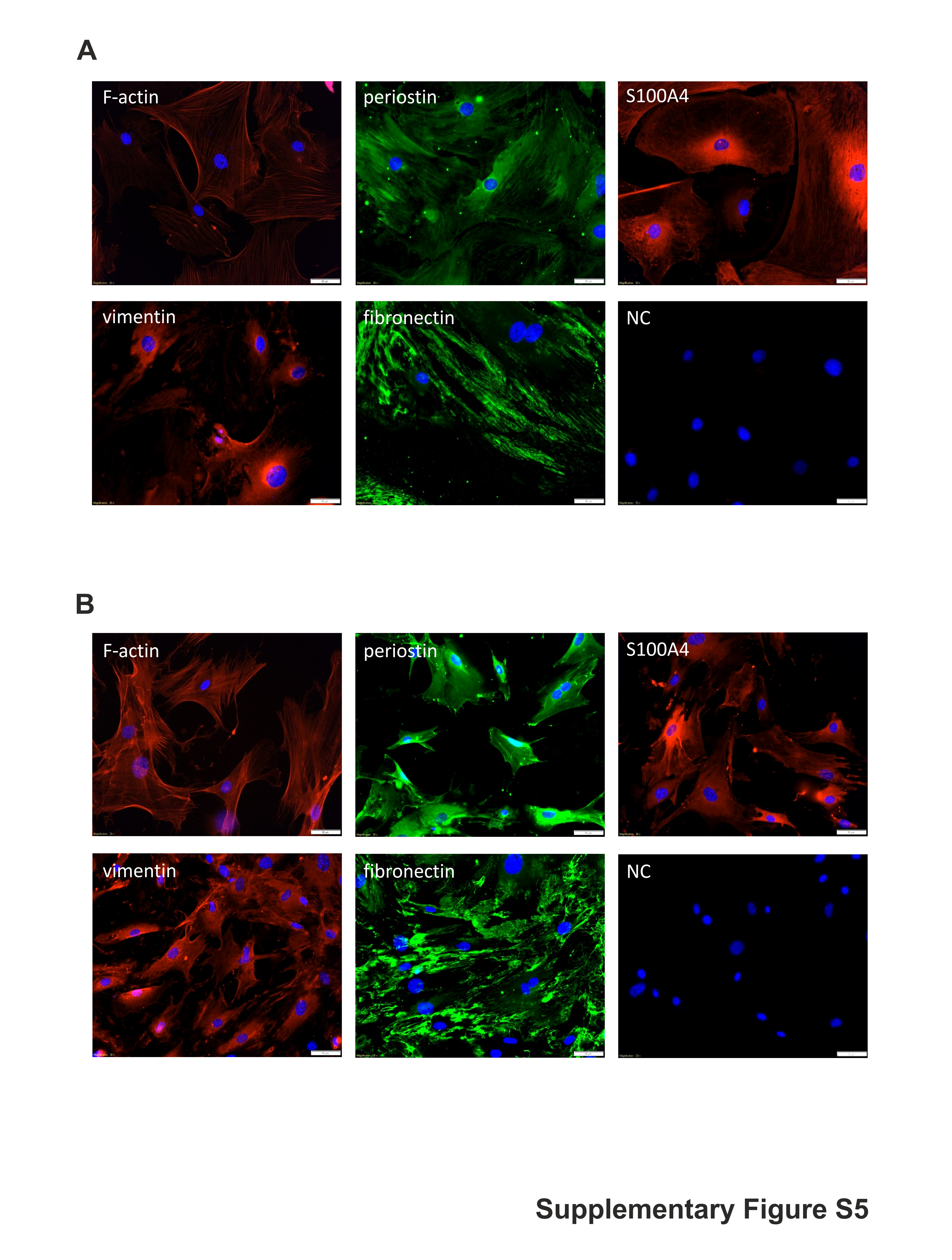
**

**
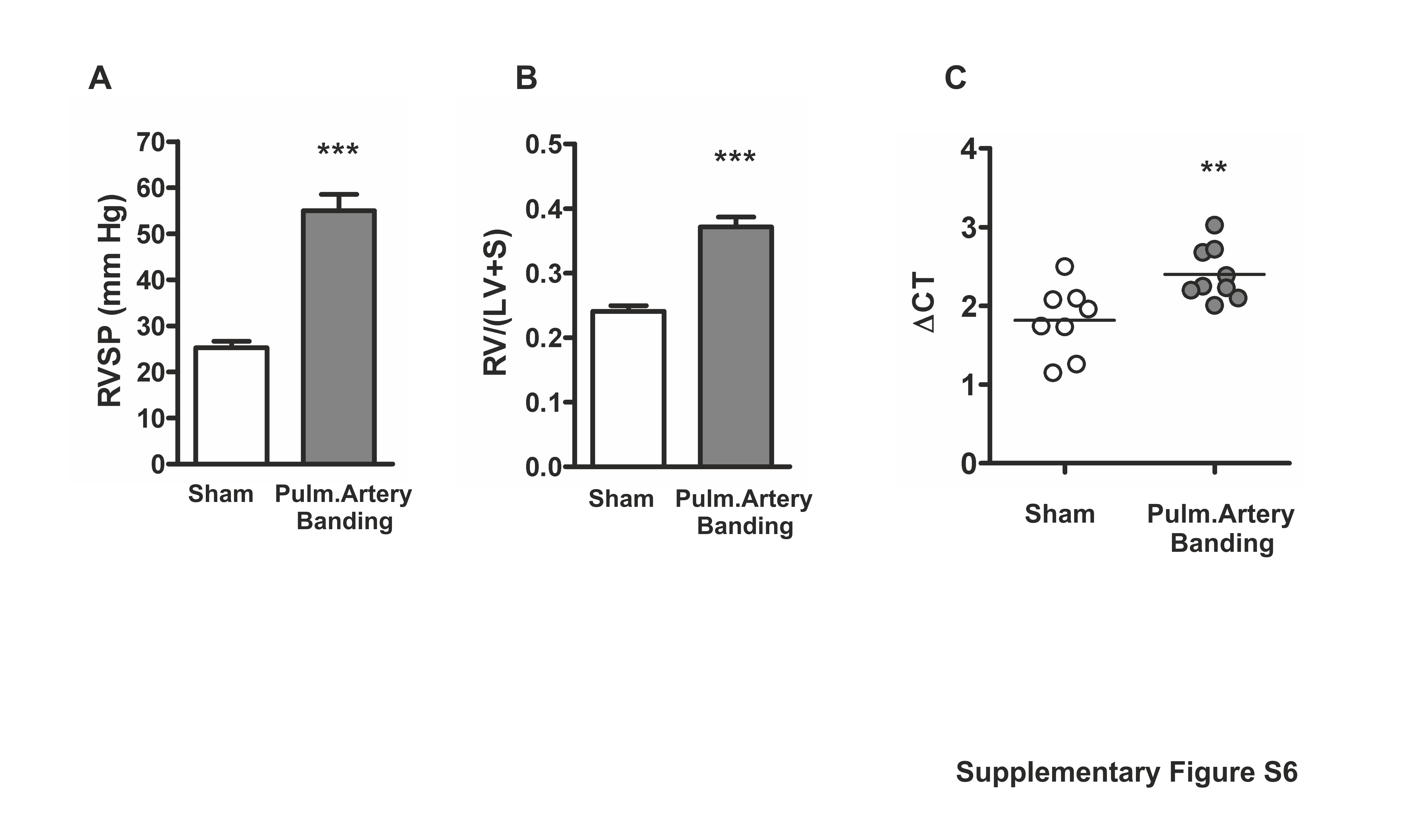
**

**
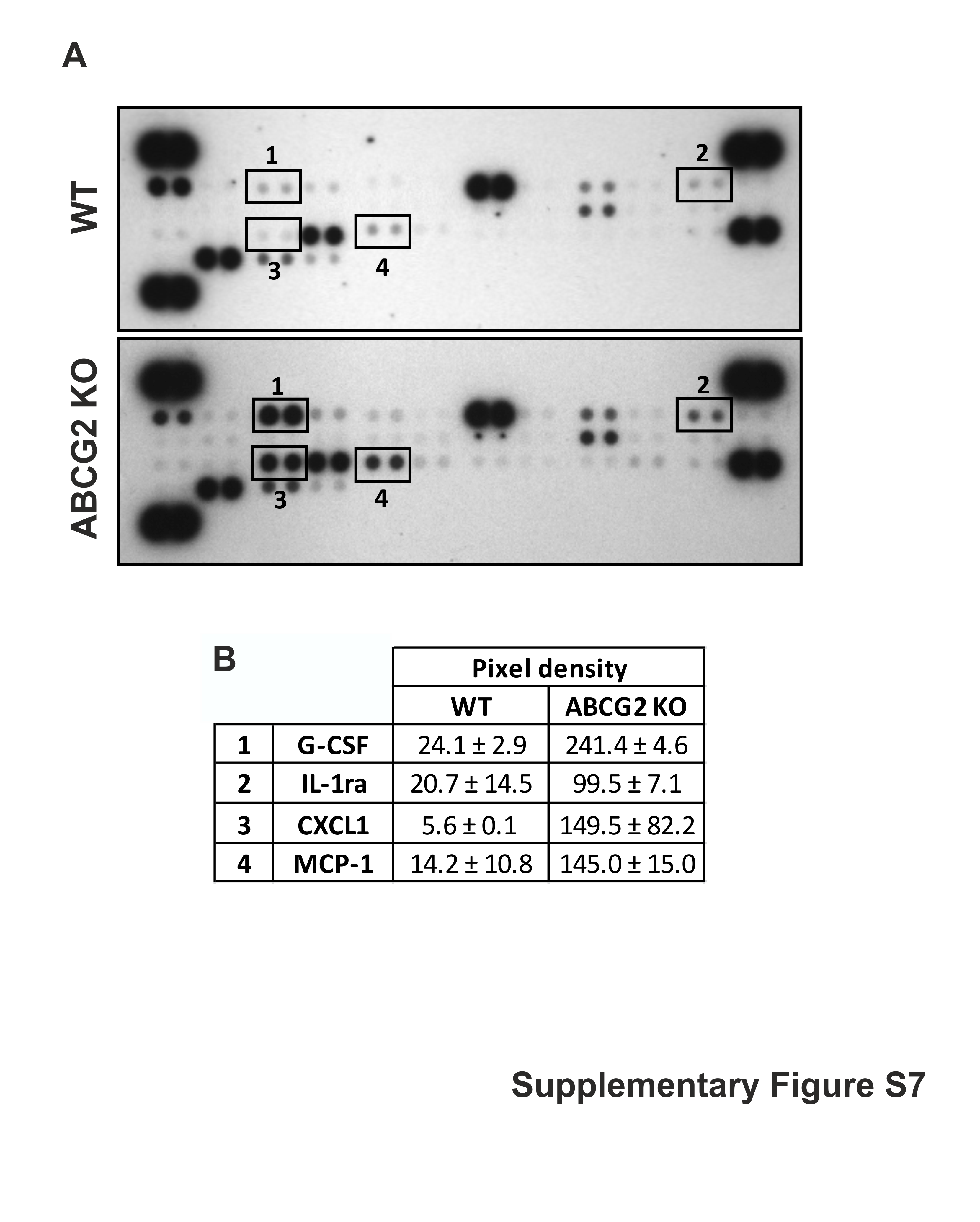
**

| **Supplemental Table 2** | | | |
| --- | --- | --- | --- |
| **Statistical results - Adjusted rank transformed ANOVA** | | | |
| **Pulmonary hypertension – related parameters** | | | |
|  | **Figure** | **Parameter** | **Significance** |
| Interaction | Fig. 1 A | RVSP | p = 0.424 |
| Genotype |  |  | p = 0.553 |
| Hypoxia |  |  | p < 0.001 |
| n numbers: | WT/nox:9, WT/HOX:9, KO/nox:10, KO/HOX:11 | | |
| Interaction | Fig. 1 B | LVSP | p = 0.759 |
| Genotype |  |  | p = 0.501 |
| Hypoxia |  |  | p = 0.037 |
| n numbers: | WT/nox:8, WT/HOX:8, KO/nox:5, KO/HOX:7 | | |
| Interaction | Fig. 1 C | SBP | p = 0.609 |
| Genotype |  |  | p = 0.712 |
| Hypoxia |  |  | p = 0.064 |
| n numbers: | WT/nox:9, WT/HOX:9, KO/nox:5, KO/HOX:11 | | |
| Interaction | Fig. 1 D | RV/LV+S | p = 0.419 |
| Genotype |  |  | p = 0.094 |
| Hypoxia |  |  | p < 0.001 |
| n numbers: | WT/nox:9, WT/HOX:10, KO/nox:11, KO/HOX:11 | | |
| Interaction | Fig. 1 E | RW/BW | p = 0.411 |
| Genotype |  |  | p = 0.265 |
| Hypoxia |  |  | p < 0.001 |
| n numbers: | WT/nox:9, WT/HOX:10, KO/nox:11, KO/HOX:11 | | |
| Interaction | Fig. 1 F | Hct | p = 0.705 |
| Genotype |  |  | p = 0.694 |
| Hypoxia |  |  | p < 0.001 |
| n numbers: | WT/nox:7, WT/HOX:7, KO/nox:7, KO/HOX:7 | | |
|  |  |  |  |
|  |  |  |  |

| **Right ventricular hemodynamic parameters** | | | |
| --- | --- | --- | --- |
|  | **Figure** | **Parameter** | **Significance** |
| Interaction | Fig. 2 B | RVEDP | p < 0.001 |
| Genotype |  |  | p = 0.088 |
| Hypoxia |  |  | p < 0.001 |
| n numbers: | WT/nox:7, WT/HOX:7, KO/nox:7, KO/HOX:7 | | |
| Interaction | Fig. 2 C | Tau index | p = 0.631 |
| Genotype |  |  | p = 0.079 |
| Hypoxia |  |  | p = 0.883 |
| n numbers: | WT/nox:6, WT/HOX:7, KO/nox:7, KO/HOX:7 | | |
| nteraction | Fig. 2 D | mindP/dt | p = 0.444 |
| Genotype |  |  | p = 0.541 |
| Hypoxia |  |  | p < 0.001 |
| n numbers: | WT/nox:7, WT/HOX:7, KO/nox:7, KO/HOX:7 | | |
| Interaction | Fig. 2 E | maxdP/dt | p = 0.944 |
| Genotype |  |  | p = 0.588 |
| Hypoxia |  |  | p = 0.003 |
| n numbers: | WT/nox:7, WT/HOX:7, KO/nox:7, KO/HOX:7 | | |
|  |  |  |  |
| **Left ventricular hemodynamic parameters** | | | |
|  | **Figure** | **Parameter** | **Significance** |
| Interaction | Fig. 3 B | LVEDP | p = 0.110 |
| Genotype |  |  | p = 0.974 |
| Hypoxia |  |  | p = 0.001 |
| n numbers: | WT/nox:5, WT/HOX:7, KO/nox:5, KO/HOX:6 | | |
| Interaction | Fig. 3 C | Tau index | p = 0.165 |
| Genotype |  |  | p = 0.922 |
| Hypoxia |  |  | p = 0.267 |
| n numbers: | WT/nox:5, WT/HOX:7, KO/nox:5, KO/HOX:6 | | |
| Interaction | Fig. 3 D | mindP/dt | p = 0.716 |
| Genotype |  |  | p = 0.471 |
| Hypoxia |  |  | p = 0.013 |
| n numbers: | WT/nox:5, WT/HOX:7, KO/nox:5, KO/HOX:6 | | |
| Interaction | Fig. 3 E | maxdP/dt | p = 0.807 |
| Genotype |  |  | p = 0.753 |
| Hypoxia |  |  | p = 0.084 |
| n numbers: | WT/nox:5, WT/HOX:7, KO/nox:5, KO/HOX:6 | | |
|  |  |  |  |
|  |  |  |  |
| **Tissue fibrosis** | | | |
|  | **Figure** | **Parameter** | **Significance** |
| Interaction | Fig. 4 B | RV Fibrosis score | p = 0.036 |
| Genotype |  |  | p = 0.216 |
| Hypoxia |  |  | p = 0.009 |
| n numbers: | WT/nox:6, WT/HOX:7, KO/nox:7, KO/HOX:7 | | |
| Interaction | Fig. 4 D | LV Fibrosis score | p = 0.002 |
| Genotype |  |  | p < 0.001 |
| Hypoxia |  |  | p < 0.001 |
| n numbers: | WT/nox:6, WT/HOX:7, KO/nox:7, KO/HOX:7 | | |
| Interaction | Fig. 4 F | Lung Fibrosis score | p = 0.847 |
| Genotype |  |  | p = 0.106 |
| Hypoxia |  |  | p = 0.698 |
| n numbers: | WT/nox:9, WT/HOX:10, KO/nox:11, KO/HOX:11 | | |
|  |  |  |  |
| **Human cardiacfibroblasts** | | | |
|  | **Figure** | **Parameter** | **Significance** |
| Interaction | Fig. 6 C | Proliferation | p = 0.926 |
| Silencing |  |  | p = 0.780 |
| Hypoxia |  |  | p = 0.780 |
| n numbers: | n:4 | | |
| Interaction | Fig. 6 D | Collagen production | p = 0.040 |
| Silencing |  |  | p = 0.008 |
| Hypoxia |  |  | p = 0.118 |
| n numbers: | n:5 | | |
|  |  |  |  |
| **Heart rate** | | | |
|  | **Figure** | **Parameter** | **Significance** |
| Interaction | Suppl. Fig. 1 | Heart rate | p = 0.533 |
| Genotype |  |  | p = 0.028 |
| Hypoxia |  |  | p = 0.783 |
| n numbers: | WT/nox:6, WT/HOX:7, KO/nox:7, KO/HOX:7 | | |
|  |  |  |  |
| **Capillary density** | | | |
| Interaction | Suppl. Fig. 3 B | RV Capillary density | p = 0.057 |
| Genotype |  |  | p = 0.752 |
| Hypoxia |  |  | p = 0.951 |
| n numbers: | WT/nox:7, WT/HOX:7, KO/nox:7, KO/HOX:7 | | |
| Interaction | Suppl. Fig. 3 D | LV Capillary density | p = 0.716 |
| Genotype |  |  | p = 0.822 |
| Hypoxia |  |  | p = 0.129 |
| n numbers: | WT/nox:7, WT/HOX:7, KO/nox:7, KO/HOX:6 | | |
